# Rhabdomyolysis Secondary to Bee Sting

**DOI:** 10.1155/2013/258421

**Published:** 2013-03-27

**Authors:** Okhan Akdur, Serdar Can, Göksu Afacan

**Affiliations:** Department of Emergency Medicine, Canakkale Onsekiz Mart University Medical Faculty, 17100 Canakkale, Turkey

## Abstract

Insect stings belonging to Hymenoptera defined as wasps, yellow jackets, bees, or hornets by human usually result in unserious clinical pictures that go with pain. Rhabdomyolysis following a bee sting is a rare condition. This paper emphasizes “rhabdomyolysis” as a rare complication of this frequently observed envenomation. Rare but severe clinical results may occur due to multiple bee stings, such as intravascular hemolysis, rhabdomyolysis, acute renal insufficiency, and hepatic dysfunction. In bee stings as in our case, clinicians should be alert for rhabdomyolysis in cases with generalized body and muscle pain. Early onset alkaline diuresis and management in patients with rhabdomyolysis are vital in protecting the renal functions and preventing morbidity and mortality.

## 1. Introduction

Insect stings belonging to Hymenoptera defined as wasps, yellow jackets, bees, or hornets by human usually result in unserious clinical pictures that go with pain [1]. On the other hand, emergency unit physicians also know that it may cause death by anaphylaxis. However, Hymenoptera stings may result in a wide range of clinical spectra ranging from localized pain to systemic reaction and organ dysfunction and multiple organ failure [2]. Rhabdomyolysis following a bee sting is a rare condition. This paper emphasizes “rhabdomyolysis” as a rare complication of this frequently observed envenomation.

## 2. Case Presentation

A 34-year-old healthy farmer was stung three times by a bee: two stings behind right ear and one on the right side of face within a 24-hour period in his working area. On the first day, he had no complaints other than pain and swellings, until when he presented to the emergency unit with fatigue and pain in the whole body on the second day. In his medical history, he had no allergic reactions, drug use, or chronic disease. On physical examination, he was conscious, general status was good, systolic blood pressure was 110 mmHg and diastolic blood pressure was 80 mmHg, his pulse was 88/min., body temperature was 36.8°C, and O_2_ saturation was 98. He had no rash or edema. In the initial laboratory results, the white blood cell count was 13.200/*μ*L, creatine phosphokinase (CPK) was 1304 U/L (26–308 U/L), CK-MB was 39 U/L (0–25), urea was 29 mg/dL, and creatinine was 0.97 mg/dL. The urine was dark in color after the patient was catheterized. On urine analysis using sticks, the myoglobin level was +++. Intravenous saline infusion was begun and fluid management was continued with 5% dextrose and Ringer's lactate alternately in order to keep the urine output over 100 mL/h. For the diffuse body pain, intramuscular 75 mg diclofenac sodium, 1000 mg intravenous paracetamol and 75 mg pethidine HCl twice were administered. Intravenous methyl prednisolone 40 mg was administered twice a day, and 45.5 mg pheniramine hydrogen maleate was given for five days, starting intravenously twice a day. The CPK level was 4786 U/L on the second day, 3158 U/L on the third day, 1521 U/L on the fourth, and 134 U/L on the fifth day. During five days of emergency unit followup, a total of 80 mEq of NaHCO_3_ and 8000 cc fluid infusion were administered and no change in renal function tests was observed. When his pain had been completely resolved, he was discharged in good health on the fifth day of treatment.

## 3. Discussion

Wasps and bees use their ovipositors to protect themselves and their colonies. It can be predicted what many species may do in a similar situation. However, it is known that some species may attack with a bare provocation. In mild climates, particularly on hot days, these events occur more frequently [3]. In this case, the first sting occurred in the working area without provocation, while the second sting occurred when trying to keep the bees away. It is notable that these stings may occur without provocation.

Venoms of wasps also contain various biogenic amines. Pain occurs immediately due to venom injection. In a very short time, erythematous, mostly papular lesions accompanied by urticaria and edema of varying degrees develop [4]. If no complication develops, typical lesions regress in 4–6 hours. This picture is frequently observed in bee stings. Sometimes, extensive local reactions may last for several days [5]. Local reactions may be managed by removing the sting, analgesics, and ice applications. The efficacy of other agents like antihistamines and corticosteroids has not been proved [6].

Hymenoptera species cause severe allergic reactions more frequently than other arthropods. The rate of venom-related systemic reaction is lower than 5% and the possibility of anaphylaxis is lower than 1% [4, 6]. In such severe reactions, the use of intramuscular adrenaline (0.3 mg) followed by hydrocortisone and chlorpheniramine maleate is critical [7]. Severe allergic reactions are often considered in cases presenting to the emergency unit with bee stings and necessary precautions are taken. However, it has been mostly neglected that bee stings may result in severe clinical pictures such as rhabdomyolysis, intravascular hemolysis, liver necrosis, and thrombocytopenia. Fatality is observed secondary to cardiac arrest due to venom toxicity or renal insufficiency on the fourth and ninth days [8]. Among these, the developing mechanism of rhabdomyolysis has not been clarified completely. It is thought that the direct toxic effect of venom on muscular tissue plays a role [9]. The causes of renal insufficiency are pigment nephropathy caused by rhabdomyolysis and intravascular hemolysis or acute tubular necrosis developed by hypotension and acute interstitial nephritis thought to occur by the direct effect of venom [10]. Histamin-like active amines, serotonin, quinines, phospholipase A_2_, hyaluronidase, melitin, and apamin are responsible for the toxic effects of venom [11, 12]. These have hemolytic, neurotoxic, and vasoactive characteristics that may cause intravascular hemolysis and rhabdomyolysis [10, 13]. Therefore, in serious clinical conditions, antihistamines and steroids have beneficial effects. Optimal hydration and alkaline diuresis decrease the ischemic acute tubular necrosis and pigment nephropathy [7]. Having determined rhabdomyolysis in our case, by using these agents and administering NaHCO_3_ and fluid infusion, we protected the renal functions until the serum CK levels decreased. In cases with fulminant progressive renal insufficiency and anuria, intensive dialysis treatment is necessary. Furthermore, some case reports have stated that treatment with plasma exchange is beneficial [14]. Another remarkable and accentuated issue is that generalized pain is sometimes irresponsive to paracetamol and NSAI drugs, and the pain may require narcotic analgesics as in our case.

Other possible clinical pictures in these cases are myocardial necrosis and infarction, centrilobular necrosis of the liver, and thrombocytopenia due to direct platelet toxicity [15].

Bee and wasp stings are commonly observed in our country [16]. Emergency room visits have been reported in 9.3–5% and familial Hymenoptera allergy has been reported in 10.2%. However, in our country, reports with the severe clinical pictures stated previously consequent to bee sting are scarce [16, 17]. Therefore, this case is valuable and significant to emphasize that bee stings in our region and in this country may cause severe clinical pictures.

Rare but severe clinical results may occur due to multiple bee stings, such as intravascular hemolysis, rhabdomyolysis, acute renal insufficiency, and hepatic dysfunction. In bee stings as in our case, clinicians should be alert for rhabdomyolysis in cases with generalized body and muscle pain. Early onset alkaline diuresis and management in patients with rhabdomyolysis are vital in protecting the renal functions and preventing morbidity and mortality. It should be known that there is potential fatality when management is delayed or administered insufficiently following bee stings.
